# Multiomics Approach to Precision Sports Nutrition: Limits, Challenges, and Possibilities

**DOI:** 10.3389/fnut.2021.796360

**Published:** 2021-12-14

**Authors:** David C. Nieman

**Affiliations:** North Carolina Research Campus, Human Performance Laboratory, Department of Biology, Appalachian State University, Boone, NC, United States

**Keywords:** exercise, nutrition, genomics, metabolomics, proteomics, carbohydrates, polyphenols, immunity

## Abstract

Most sports nutrition guidelines are based on group average responses and professional opinion. Precision nutrition for athletes aims to improve the individualization of nutrition practices to optimize long-term performance and health. This is a 2-step process that first involves the acquisition of individual-specific, science-based information using a variety of sources including lifestyle and medical histories, dietary assessment, physiological assessments from the performance lab and wearable sensors, and multiomics data from blood, urine, saliva, and stool samples. The second step consists of the delivery of science-based nutrition advice, behavior change support, and the monitoring of health and performance efficacy and benefits relative to cost. Individuals vary widely in the way they respond to exercise and nutritional interventions, and understanding why this metabolic heterogeneity exists is critical for further advances in precision nutrition. Another major challenge is the development of evidence-based individualized nutrition recommendations that are embraced and efficacious for athletes seeking the most effective enhancement of performance, metabolic recovery, and health. At this time precision sports nutrition is an emerging discipline that will require continued technological and scientific advances before this approach becomes accurate and practical for athletes and fitness enthusiasts at the small group or individual level. The costs and scientific challenges appear formidable, but what is already being achieved today in precision nutrition through multiomics and sensor technology seemed impossible just two decades ago.

## Personalized and Precision Nutrition

A core tenet of the 2020–2025 Dietary Guidelines for Americans (DGAs) is that a healthy dietary pattern is a customizable framework that supports tailored individual choices to meet personal preferences (1). The DGAs have also been characterized, however, as population-level recommendations that do not provide ideal nutrition guidance for each individual (2). Individuals vary widely in their metabolic responses to specific components of the DGAs, and most of this variance is unexplained (3, 4). The National Institutes of Health (NIH) and other agencies are focused on investigations to improve scientific understanding of these individual differences, adding momentum to the paradigm shift toward personalized nutrition (5, 6). This narrative-style mini-review outlines the limits, challenges, and possibilities of personalized nutrition, with a focus on multiomics approaches and how these can be applied to sport and exercise nutrition.

Personalized nutrition is expected to grow from $8.2 billion in 2020 to $16.4 billion by 2025 (7). This growth is being driven by increasing health and fitness awareness by consumers, dramatic drops in genotyping costs, access to free public archives of genetic variation data, digital healthcare, direct-to-consumer (DTC) kits, widespread availability and use of smartphone apps, consumer demand for supplements, and an aging population (7, 8). Advocates also urge that personalized nutrition compared to traditional approaches will be more effective in motivating individuals to improve their dietary intake and thereby mitigate disease risk factors (9, 10).

Several definitions for personalized nutrition have been proposed (10, 11). The International Life Sciences Institute emphasized that personalized nutrition for the generally healthy population should use “individual-specific information, founded in evidence-based science, to promote dietary behavior change that may result in measurable health benefits” (2). Others prefer the term precision nutrition that is focused on a systems biology and multiomics approach (i.e., using the tools of genomics, transcriptomics, proteomics, metabolomics, microbiomics, epigenetics) with integration bioinformatics and machine learning to sharpen the scientific certainty needed for specific nutrition recommendations (3, 11).

Personalized and precision nutrition are emerging disciplines that aim to stratify individuals into ever-smaller groups as the science develops and specific nutrition guidance can be conveyed accurately (7, 11). There are many challenges to overcome before personalized and precision nutrition becomes an accepted component of nutrition science and professional practice. Metabolic heterogeneity with high individual-to-individual variance is largely unexplained (4). Precision nutrition aims to improve scientific understanding of responders and non-responders to dietary interventions. This process will require a tremendous investment by multiomics-focused investigators and funding agencies, with strong bioinformatics support (11, 13). Once metabolic heterogeneity is better understood, the findings must next be translated to accurate dietary advice that is efficacious and health-promoting (6, 11). The entire process will have to be linked to health behavior change support and research to determine if people are motivated enough to change and accept this costly and complex approach over the long term.

We are currently in a transition period with the rapid expansion of nutrition-based multiomics data but a lack of well-designed studies to demonstrate efficacious dietary recommendations at the small group or individual level (12). Humans have more than 21,000 genes, and each person has more than 50,000 single nucleotide polymorphisms (SNPs) (3, 13). Observed phenotypes are impacted by many genes, SNPs and other types of genetic variants, and epigenetic changes from environmental and lifestyle factors that influence the way genes work (13). People vary widely in how they respond to plant food bioactives and phytochemicals, in part due to differences in absorption, distribution, metabolism, and excretion (ADME) (6). As a result, inter-individual variation is considerable and far exceeds intra-individual variation in most multiomics studies (4, 12). For example, even among twins, gut microbiome alpha diversity (richness) varies more over time between the twins than within a twin (14). All of this complicates the translation of genomics and other multiomics data into dietary recommendations for small groups and individuals (13).

A new generation of studies is needed with in depth phenotyping and integration of multiomics data with machine learning (a subbranch of Artificial Intelligence) to aid in the development of predictive precision nutrition models (6, 11, 15). Supervised and unsupervised machine learning algorithms focus on patterns within large and complex precision nutrition datasets to develop maximum likelihood predictions about the outcomes of interest (15). The use of machine learning in precision nutrition is an emerging discipline, and one of the fundamental challenges is the development of high-quality datasets from large cohorts from which pertinent measurements have been obtained. Another challenge is the use of evaluation metrics to verify the actual effectiveness of the prediction models (15).

Decades of research on the genetic risk for obesity can serve as a lesson for the challenges that lie ahead in precision nutrition. The genetic component of BMI in the population accounts for about 40 to 50% of its variance after adjustment for age and sex, providing room for modifying effects of genetic variation to be assessed (16). It is now apparent from genome-wide association studies (GWAS) combined with large SNPs panels that obesity genetic risk is shaped by hundreds, perhaps thousands of DNA variants (16). As a result, no genetically based clinical screening algorithm has attained the predictive power needed to calculate obesity risk for individuals (16). The most important message from obesity genetics research is that people do not all have the same proneness to becoming obese and despite decades of effort this still remains difficult to predict.

What does this mean for precision nutrition? Both obesity and the way people respond to dietary interventions are impacted by many intrinsic and extrinsic factors. Genomics is just one of many components to measure and consider. Novel precision nutrition programs to manage obesity have been designed with personalized macronutrient compositions that vary based on the individual's genotype, enterotype, and other related factors (17). To refine this approach, comprehensive data sets from large groups are needed that include demographics, anthropometry, diet intake, physical activity, genomics, transcriptomics, epigenetics, proteomics, metabolomics, and environmental exposure (3, 11, 12). With machine learning, these types of data sets can be modeled to improve understanding of inter-individual variation and the development of more accurate precision nutrition recommendations. Although remarkable progress has been made, multiomics-based solutions and elucidations remain a work in progress, with high expectations that this will be a successful, albeit costly initiative (6, 13).

## Precision Nutrition for Athletes and Physically Active Individuals

So what insights can be applied from precision nutrition to athletes and physically active individuals? Athletes vary widely in their lifestyle habits and metabolic and physiological responses to sports foods and supplements (18, 19). Most sports nutrition guideline statements and periodized nutrition plans are based on group average responses and professional opinion (20–22). Several organizations have recommended a biology systems-based approach to adapt sports nutrition guidelines for a more personalized approach (19–23).

**Figure 1:**
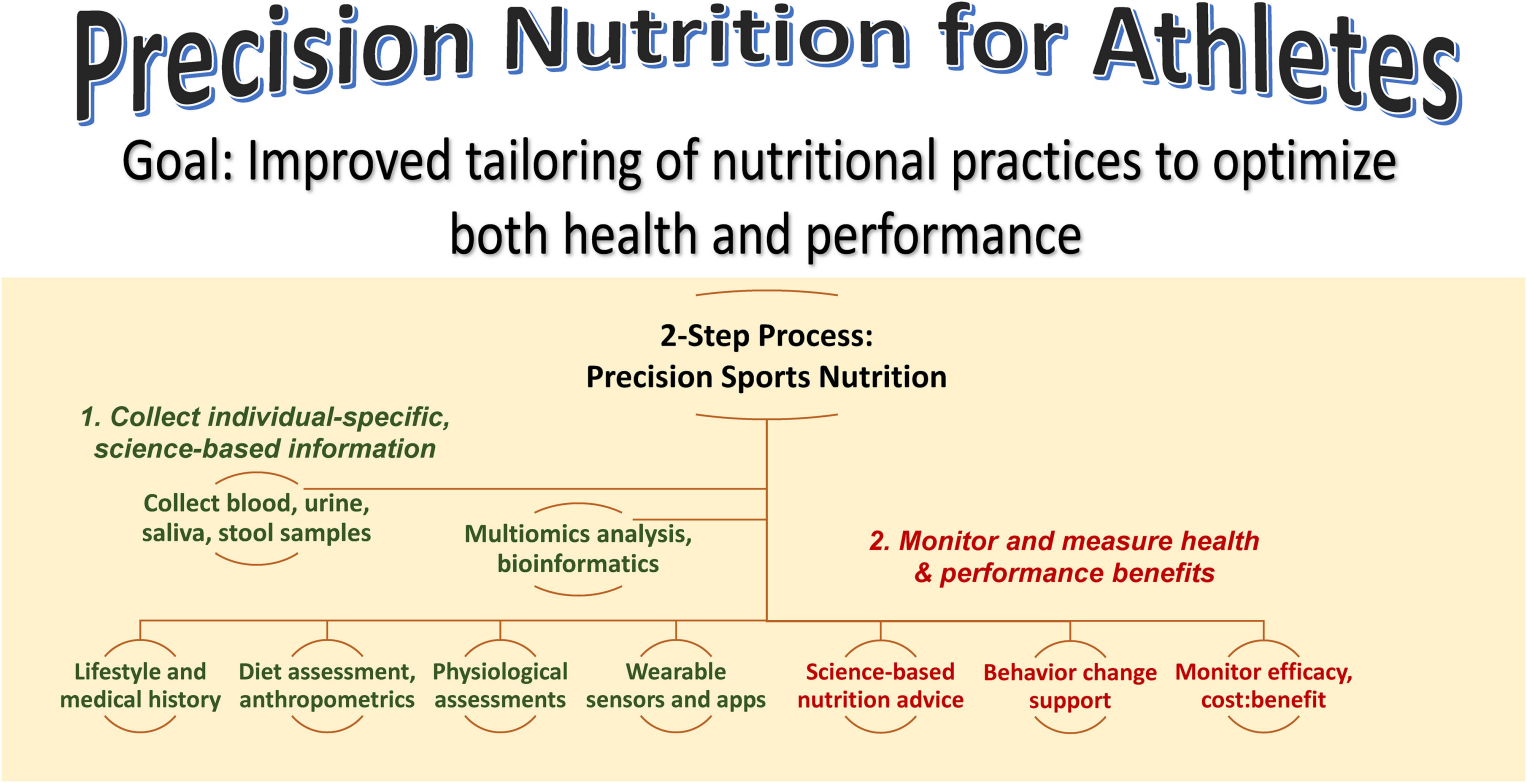
Precision nutrition for athletes aims to improve tailoring of nutrition practices to optimize both health and performance. This is a 2-step process that emphasizes 1) the acquisition of individual-specific, science-based information using a variety of sources and 2) delivery of science-based nutrition advice with behavior change support and monitoring of efficacy and benefits relative to cost.

Figure 1 summarizes a precision nutrition approach for athletes. This is a 2-step process that focuses on collecting as much individual-specific, science-based information as possible on the individual, and then developing and delivering individualized nutrition guidance while monitoring and measuring health and performance outcomes. The first step emphasizes the acquisition of information using a variety of sources including lifestyle and medical histories, dietary assessment, physiological assessments from the performance lab and wearable sensors, and multiomics data from blood, urine, saliva, and stool samples. Depending on resources and during this transition period of continued investigation by scientists, this step may involve just a few targeted biomarker data with high effect sizes. The second step involves the delivery of science-based nutrition advice, behavior change support, and the monitoring of health and performance efficacy and benefits relative to cost. The sports nutrition precision approach is an emerging science with years of additional studies of large groups needed to ensure accurate, practical, and individualized nutrition guidance.

Despite scientific reservations at this point in time, the precision sports performance and nutrition DTC companies have already moved forward into the marketplace. A growing number of DTC companies gather pertinent demographics and lifestyle data, extract DNA from consumer's saliva samples using home kits, assess the presence or absence of genetic variants, predict inherent athletic ability, and/or provide individualized recommendations on exercise training and diet intake to improve performance (8, 24). Bloodwork and DNA data are also used to individualize guidelines for building muscle, reducing inflammation, boosting energy, optimizing mood, raising metabolism, improving cognition, and maintaining bone health.

The current consensus among genetics researchers is that DTC genetic tests are unable to provide accurate information regarding early athletic talent, training recommendations to maximize performance, or how to lose fat and build muscle (25, 26). Often the DTC genetic tests focus on polymorphisms for just a few genes (e.g., alpha-actinin-3 or ACTN3, and angiotensin-converting enzyme or ACE) despite the fact that there is no consistent scientific evidence that they have a sizeable influence on complex attributes such as athletic performance (8, 24–26). Hereditability estimates for cardiorespiratory fitness or VO2max are relatively high (about 50%) (27, 28). Nonetheless, studies linking genotype and SNPs to cardiorespiratory fitness and other related attributes such as exercise training responses and exercise-induced changes in cardiometabolic risk factors have failed to produce definitive panels that could be used by DTC companies (27, 29–31). The use of global metabolomics and proteomics profiling as correlates of cardiorespiratory fitness has only recently been explored and these are best described as preliminary studies (32, 33).

Common nutrition-related traits that are assessed by DTC companies include food intolerance (e.g., lactose intolerance), food sensitivities (i.e., caffeine sensitivity), macronutrients (e.g., lipid metabolism), micronutrient metabolism (e.g., vitamins D and C metabolism), eating behavior (e.g., weight management), and oxidative stress (antioxidant and detoxifying capacity) (8). DTC companies seldom provide information on the specific genetic variants that are being assessed, and when listed, are often unrelated to the trait (8). A growing number of gene-nutrient interactions that may influence sport performance have been proposed, but in general, there is a dearth of strong research data at this time to make recommendations for athletes based on nutrigenomics (31).

Caffeine is one of just five evidence-based dietary supplements linked to performance optimization (18, 21), and serves as a prime example of the challenges in sport nutrigenomics. Studies generally support caffeine ingestion (3–13 mg/kg) for improved performance in a broad range of exercise modes, but individual responses vary widely (34). The cytochrome P450 1A2 (CYP1A2) gene produces an enzyme that metabolizes caffeine, but more than 13 SNPs can modify this gene's metabolic activity (35). One of the CYP1A2 SNPs is rs762551, and A/C and C/C carriers (54% of population) experience decreased caffeine metabolism activity in comparison to more rapid metabolism in A/A carriers (46% of population). Although CYP1A2 polymorphism has been hypothesized to alter caffeine's ergogenic effect, studies thus far are mixed, and CYP1A2 genotyping as a service to athletes is not recommended until more is known (35, 36).

Another interesting nutrient that serves as an example of the challenges that lie ahead in sport nutrigenomics is choline. Choline is an essential nutrient involved in multiple biochemical pathways related to performance including acetylcholine production for neurotransmission and phosphatidylcholine formation in muscle membranes (37, 38). Low dietary intake of choline can diminish concentrations of phosphatidylcholine in muscle cell membranes, making them more fragile and prone to exercise-induced muscle damage and leakage of creatine phosphokinase (CPK) (39). Common polymorphisms of genes involved with choline metabolism (about 10% of the population) include SNPs (rs2771040, rs1557502) associated with the solute carrier 44 choline transporter member 1 (SLC44A1) and choline kinase (CHKB) genes (38, 39). People who carry these SNPs develop extremely high serum creatine phosphokinase (CPK) levels after stressful exercise bouts that include eccentric contractions (e.g., downhill running). These individuals may also develop rhabdomyolysis when dietary patterns low in choline are combined with stressful levels of exercise training. Some advocate widespread SNP screening to identify these individuals combined with recommendations to increase choline (e.g., more eggs and liver) and methyl-folate intake to mitigate exercise-induced muscle damage (39–41). However, definitive research to support this strategy is lacking, and long-term health and performance effects of carrying these gene polymorphisms are unknown. Studies have consistently failed to demonstrate that supplemental choline has a positive influence on physical or cognitive performance (42, 43).

Precision nutrition relies on what can be accurately assessed at the individual level, and this process often begins and is ultimately limited with assessment of dietary intake and physical activity. The IOC recommends that a complete nutritional assessment of the athlete's diet should be undertaken before decisions regarding supplement use are made (18). Data obtained from dietary intake assessments (e.g., 24-h recall, food frequency questionnaires, dietary records) have limited utility because they are burdensome and based on self-report, memory, social desirability reporting bias, and behavior change reactivity to monitoring (44, 45). Currently, physical activity compared to dietary intake monitoring tools are more advanced (44). Technological advances in wearable sensors have improved physiological and performance monitoring of mental and physical stress, sleep quality, blood glucose levels, body temperature, hydration status, oxygen consumption, heart rate, blood pressure, and exercise workloads (46). The use of wearable sensors facilitates continuous and real-time tracking of targeted outcomes that should strengthen the usefulness of nutrition guidance down to the individual level. The use of multimodal wearable sensors and miniature cameras in the area of nutritional assessment is expanding rapidly (44, 45, 47). The ultimate goal is to make physical activity, dietary intake, and physiological monitoring seamless, passive, accessible, and accurate, and this will transform the effectiveness of precision nutrition (46–48).

## Systems Biology Approach to Precision Sports Nutrition

Vigorous acute exercise bouts increase body metabolic demands 6 to 20 times above resting levels, and have profound, transitory effects on gene expression and blood/tissue levels of numerous metabolites, lipid mediators, and proteins (49–59). These exercise-induced molecular changes are complex and incompletely understood. Human systems biology approaches with integrated multiomics profiling have been initiated to improve scientific understanding of the molecular underpinnings of related health and disease prevention benefits (50).

Multiomics profiling in exercise science has expanded rapidly due to the development of new technologies that provide simultaneous measurement of hundreds and thousands of molecules from small amounts of body fluids, cells, and tissues. Recent studies have linked acute vigorous exercise to changes in about 6,000 transcripts (from RNA sequencing), more than 300 proteins, and 300 to 700 metabolites (49–56). Integrated molecular profiling during recovery from vigorous exercise indicates that multiple biological processes are affected including energy metabolism, oxidative stress, immune function and inflammation, tissue repair and remodeling, signaling pathways, cell growth and mobility, cardiovascular signaling and angiogenesis, and apoptosis (49–52). Exercise proteomics has shown that hundreds of proteins are secreted by the muscle and other tissues discretely or within extracellular vesicles to regulate physiological processes throughout the body (54, 55, 60). Exercise metabolomics has established that following intensive bouts lasting more than two hours, large-fold changes in numerous and diverse lipid-related metabolites occur reaching their nadir within a few hours with abatement after one day of recovery (53, 58). Other exercise-induced plasma metabolite shifts include a variety of amino acids and tricarboxylic acid (TCA) cycle intermediates including malate, aconitate, citrate, fumarate, succinate, and alpha-ketoglutarate. Postexercise changes in amino acids support metabolic requirements, and increases in TCA metabolites facilitate regulation of inflammation and immune function (50, 51, 53, 56). Intensive and prolonged exercise also increases plasma and muscle levels of 50 to 100 bioactive oxidation products from polyunsaturated fatty acids called oxylipins (57). Oxylipins have vital regulatory roles in many physiological processes including immune function, inflammation, cardiac and vascular function, and blood coagulation (57).

Thus, thousands of molecules are transiently affected by acute exercise, but the complex interplay between these molecules, the wide range of responses measured between individuals, and the use of relatively small sample sizes have limited scientific consensus (49–52). Fewer molecules differentiate exercise trained and untrained states compared to the much larger but transient shifts in proteins and metabolites following acute exercise bouts, and there is little overlap (49–52). Thus, a summation effect from regular acute exercise-induced shifts in gene expression and thousands of proteins and metabolites may play a larger role in mediating health effects than with chronic adaptations. The Molecular Transducers of Physical Activity Consortium (MoTrPAC) was established through an NIH Common Fund program to expand the science in this area and generate a molecular map of acute and chronic exercise training (61).

The application of multiomics approaches to sports nutrition is still an emerging area of scientific endeavor. Global and targeted metabolomics (with lipidomics) and proteomics improves the capacity to capture the complex biochemical effects resulting from a nutritional intervention with athletes during an exercise bout (62). Seminal studies in this area indicate that postexercise increases in lipid-related metabolites, oxylipins, and inflammatory cytokines after hours of intensive cycling are strongly mitigated when overnight-fasted athletes ingest carbohydrate compared to water only (62–65). Exercising under “low-carbohydrate” availability is physiologically stressful with widespread gene expression, cell signaling, inflammation, immune system activation, and elevated oxylipin production. This approach has been posited as advantageous for training adaptations, but there is little scientific support linking “training carbohydrate low” with improved exercise performance over the long term (62).

Polyphenol ingestion as a countermeasure to exercise-induced inflammation is receiving increasing attention by investigators (62, 63, 66). Earlier studies reported few discernable benefits of increased polyphenol intake for athletes, but research design deficiencies portrayed a misunderstanding of polyphenol bioavailability and metabolism, effective dosing protocols, and appropriate outcome measures to capture bioactive effects (62). A recent study showed that adding 1 cup of blueberries per day for two weeks prior to a 75-km cycling time trial strongly attenuated post-exercise plasma levels of 10 proinflammatory oxylipins (63). The cyclists ingesting blueberries experienced a 14-fold variation, however, in plasma levels of 24 blueberry gut-derived metabolites following supplementation. The highest gut phenolic responders to blueberry intake experienced the lowest post-exercise plasma oxylipin levels. Little is known regarding the reasons for the high inter-individual variation in gut-derived metabolites after polyphenol ingestion. This may be related in part to differences in gut microbiota α-diversity (richness) and differences in phase I and II metabolic enzymes and phase III transporters (14, 67). Whether or not the cyclists with a low gut-phenolic response to 1 cup/day blueberry ingestion would benefit from doubling their intake is unknown.

The gut microbiota includes a large collection of bacteria, viruses, fungi, and archaea, and the richness or α-diversity varies widely between individuals (14, 68). Plant-based dietary patterns and regular exercise training have an influence on the gut microbiota, increasing α-diversity and the production of metabolites such as short chain fatty acids (SCFA) from dietary fiber and gut-derived phenolics from plant polyphenols (14, 68, 69). SCFAs may support athletic performance by influencing fuel utilization and skeletal muscle function, and gut-derived phenolics may improve post-exercise metabolic recovery by mitigating inflammation (68). SCFAs are the preferred fuel for colonocytes, and have also been linked to regulation of energy homeostasis, body weight, immune function, and inflammation (69).

## The Future of Precision Sports Nutrition

The metabolic variance in the way individuals respond to exercise and nutritional interventions is considerable and largely unexplained. The other major challenge in precision nutrition includes the translation of costly multiomics biomarker data to evidence-based individualized nutrition recommendations that are acceptable to the athlete and efficacious in terms of actual performance, recovery, and health.

At this time, a precision sports nutrition approach based on integrated multiomics to tailor recommendations at the individual athlete level is an emerging discipline with more questions than answers. Precision nutrition emphasizes multiomics tools and methods to sharpen the scientific certainty needed for specific nutrition recommendations for athletes and fitness enthusiasts. However, to make this work, larger studies are needed that focus on mechanisms underlying metabolic heterogeneity with deep phenotyping, multiomics, and machine learning (6). Thus, precision nutrition will require huge investments and scientific advances before this approach becomes accurate and practical for athletes. The costs and scientific challenges make this stratagem appear unattainable, but what is being accomplished today in precision nutrition seemed impossible just two decades ago.

## Author Contributions

DN wrote this manuscript and agrees to be accountable for the content of the work.

## Conflict of Interest

The author declares that the research was conducted in the absence of any commercial or financial relationships that could be construed as a potential conflict of interest.

## Publisher's Note

All claims expressed in this article are solely those of the authors and do not necessarily represent those of their affiliated organizations, or those of the publisher, the editors and the reviewers. Any product that may be evaluated in this article, or claim that may be made by its manufacturer, is not guaranteed or endorsed by the publisher.
